# A novel mask to prevent aerosolized droplet dispersion in endoscopic procedures during the coronavirus disease pandemic

**DOI:** 10.1097/MD.0000000000026048

**Published:** 2021-07-02

**Authors:** Tadateru Maehata, Hiroshi Yasuda, Hirofumi Kiyokawa, Yoshinori Sato, Masaki Yamashita, Yasumasa Matsuo, Kazunari Nakahara, Hiroyuki Yamamoto, Fumio Itoh

**Affiliations:** Division of Gastroenterology and Hepatology, Department of Internal Medicine, St. Marianna University School of Medicine, Kawasaki, Japan.

**Keywords:** aerosol droplet, coronavirus disease, endoscopy, microdroplet dispersion, SARS-CoV-2

## Abstract

Endoscopic procedures increase the risk of transmission of severe acute respiratory syndrome coronavirus 2 to medical staff, because aerosols are generated during upper gastrointestinal endoscopy. There have been several reported studies on devices for infection prevention; however, few reports have validated them. Therefore, we developed a novel mask to prevent the diffusion of aerosol droplets from patients undergoing endoscopy.

We compared microdroplet dispersion during coughing episodes when using the novel mask with microdroplet dispersion when using the conventional mouthpiece alone.

The mean number of microdroplets was significantly smaller in the group that used the novel mask (57.9 ± 122.91 vs 933.6 ± 119.80 droplets; *P* = .01).

The novel mask may aid in reducing the degree of exposure of medical personnel to microdroplets and the risk of subsequent infection.

## 1 Introduction

As of August 2, 2020, the coronavirus disease (COVID-19) pandemic, caused by the severe acute respiratory syndrome coronavirus 2 (SARS-CoV-2), had affected more than 17.64 million people; the confirmed global death toll was 680,000. COVID-19 is mainly transmitted through respiratory droplets or by direct contact.^[1]^ Thus, it primarily spreads when people who have contracted the viral cough, sneeze, or retch. Upper endoscopic procedures, including esophagogastroduodenoscopy, endoscopic ultrasonography, and endoscopic retrograde cholangiopancreatography, can increase the risk of SARS-CoV-2 transmission to endoscopists and their assistants; this is because coughing and retching, which subsequently generate aerosols, can occur during upper gastrointestinal endoscopy.^[2]^ Some devices that prevent the diffusion of aerosol droplets from patients undergoing endoscopy have been developed^[3–5]^; however, the efficacy of these devices has not been validated. Therefore, we developed and verified the efficacy of a novel device intended to prevent the diffusion of aerosol droplets from patients undergoing endoscopy.

## 2 Materials and methods

### 2.1 Device description

**Figure 1:**
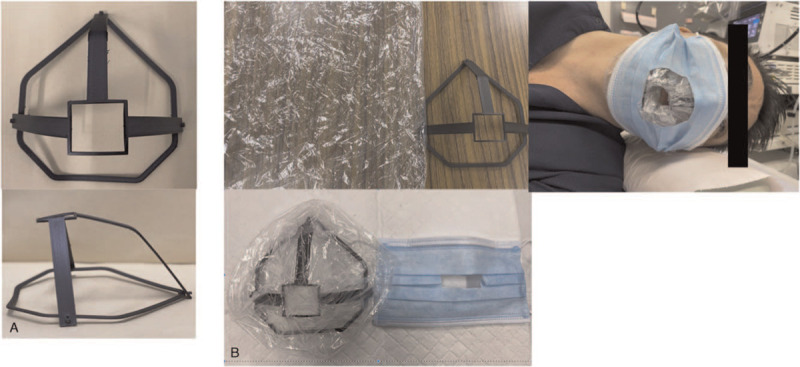
Photograph showing the novel mask. (A) An image showing the 3-dimensional (3D)-printed frame. (B) Image showing the 3D-printed frame with an off-the-shelf, A4-sized, clear, plastic bag.

We designed a novel facemask using a 3-dimensional (3D) printer comprising a 3D-printed frame, an off-the-shelf A4-sized clear plastic bag, and an off-the-shelf facemask. A hole was created at the bottom of the plastic bag for the insertion of the endoscope. After covering the mouth and nose of the patient with the novel mask, the latter was then covered with an off-the-shelf facemask to secure it (Fig. 1). The novel mask had a reinforced vertical middle line, which provided for a wider and more stable breathing space, allowing patients to breathe more comfortably; electronic monitoring was routinely needed during the endoscopic procedure. The 3D-printed material combined nylon and micro-carbon fibers into filaments. Therefore, the 3D-printed frames were extremely light (5.5 g) and could be worn easily. We used clear plastic bags, which being commercially available products, were made from polyethylene.

### 2.2 Aims and study design

This feasibility study intended to evaluate the efficacy of the novel mask for preventing diffusion of aerosol droplets among healthy volunteers.

We compared microdroplet dispersion during coughing episodes using the novel mask with that of using the normal mouthpiece alone, in three healthy volunteers who were medical professionals. This study was exempted from Institutional Review Board (IRB) approval after an IRB review, because it did not involve a medical device. This study was conducted according to the principles of the Declaration of Helsinki.

To assess efficacy, we evaluated the number of particles measuring > 0.5 μm that were dispersed when the volunteer coughed. We used high-definition cameras and laser lighting to examine the microdroplets that were dispersed when the volunteer coughed during an endoscopic procedure. With this technology, the visualized space was illuminated with straight lines of uniform light, and the faint scattered light emitted by dispersed particles was detected using a high-speed camera with ultra-high sensitivity and low noise characteristics; thus, these particles were visualized in real-time.

The microdroplets generated during coughing were forced into a uniform downflow, and the number of particles measuring > 0.5 μm in size that passed through the downstream counting area were counted in real-time at 30 fps (every 30 seconds) using the images obtained. We compared the average number of particles (> 0.5 μm) in 20 coughing episodes (10 episodes with the mask and 10 without it) per person.

### 2.3 Statistical analysis

As this was a feasibility study, no sample size calculations were conducted. These were paired data; therefore, each person underwent 2 measurements: “with mask” and “without mask.” We then evaluated whether the differences between “with mask” and “without mask” values were normally distributed, using the Shapiro--Wilk test; as highly significant (*P* = .005) results were observed, the assumption of normality was not met. The Wilcoxon signed-rank test was therefore employed for evaluating differences between the “with mask” and “without mask” values.

## 3 Results

**Figure 2:**
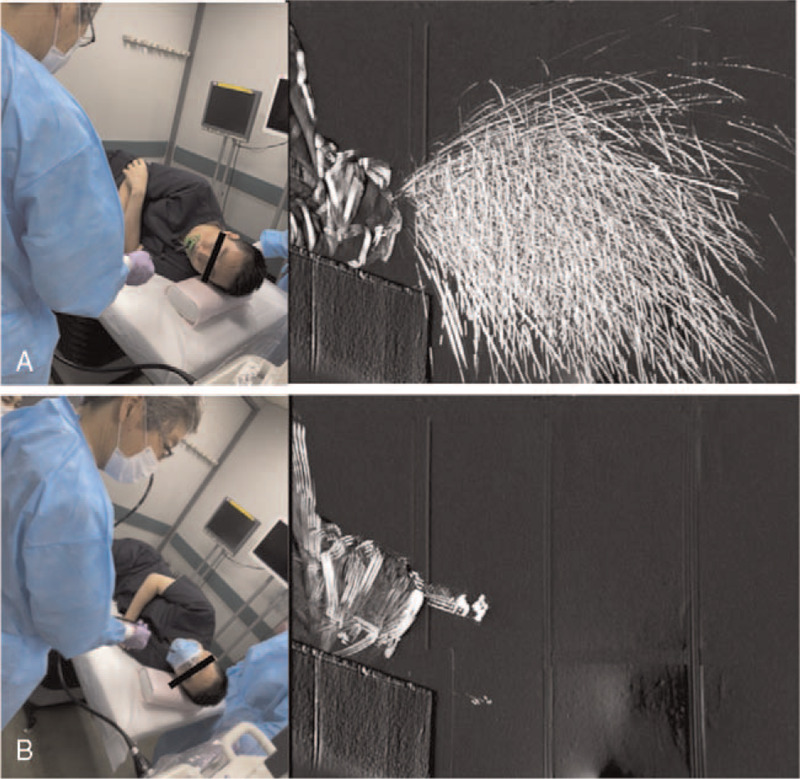
Simulation of microdroplet spread on coughing in a clean room without natural ventilation. (A) During coughing episodes without the novel mask (with the normal mouthpiece alone), the microdroplets are widely distributed in the air. (B) During coughing episodes while wearing the novel mask, almost all microdroplets are blocked.

We examined the number of microdroplets dispersed when the volunteer coughed with and without the novel mask. During coughing episodes without the mask, the microdroplets were widely distributed in the air, whereas in those with the mask, nearly all microdroplets were blocked (Fig. 2).

**Figure 3:**
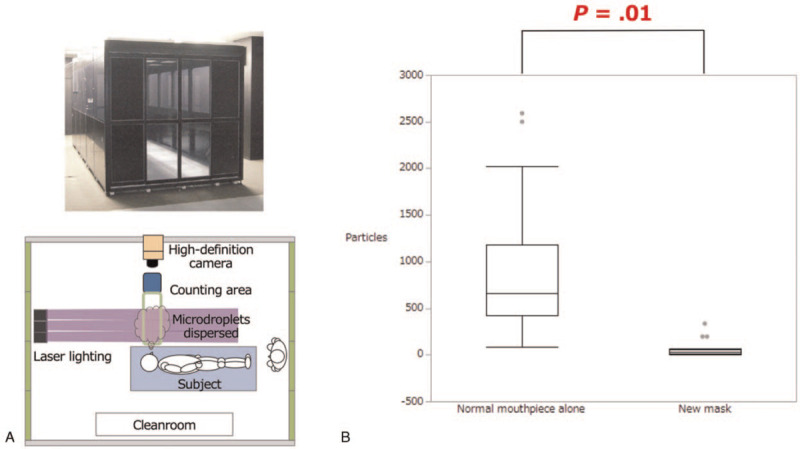
(A) Simulation of coughing to count the number of microdroplets in a cleanroom without natural ventilation. (B) Box plots showing the mean number of microdroplets counted during episodes of coughing in the group with the novel mask and in that without the novel mask.

The mean number of microdroplets was significantly smaller in the group with the novel mask than in the group without it [57.9 ± 122.91 vs 933.6 ± 119.80 droplets (*P* = .01); Fig. 3].

## 4 Discussion

This study showed that microdroplets arising because of coughing during esophagogastroduodenoscopy are widely distributed in the air. Our results suggest that microdroplet dispersion through coughing, sneezing, and retching during upper endoscopic procedures may pose a substantial risk for virus transmission to endoscopists and assistants. Furthermore, we demonstrated that the novel mask developed in this study reduces exposure of the endoscopists and assistants to microdroplets and the risk of subsequent infection.

The Centers for Disease Control and Prevention has published studies indicating that asymptomatic or presymptomatic individuals can spread SARS-CoV-2.^[6]^ Hence, the mental and physical burden upon the medical staff in endoscopy units who face a constant risk of COVID-19 infection is substantial.^[7]^

Several studies have described tools for preventing the diffusion of aerosol droplets from patients undergoing endoscopy. Marchese et al^[3]^ reported the use of an anesthetic facemask to minimize the risk of virus transmission, and Kobara et al^[4]^ reported using a shield constructed from a vinyl box. Endo et al^[5]^ devised a novel disposable system utilizing nonwoven fabric and a mouthpiece with tips for fixing a belt. However, the efficacy of these devices for preventing microdroplet dispersion has not been verified. To the best of our knowledge, ours is the first study to verify a tool for the prevention of microdroplet dispersion from patients undergoing endoscopy.

A simple and inexpensive device that prevents the diffusion of aerosol droplets from subjects is necessary during endoscopic procedures. This new mask is simple and can be made from easily available materials; the material of the 3D-printed frame is inexpensive (approximately 1 US dollar). Furthermore, as the device is created using a 3D printer, it is adjustable in size and can be used in bronchoscopy and transesophageal echocardiography. A nasal cannula delivering supplemental oxygen and suction tubes can also be inserted from the side.

This study has several limitations. First, it involved a small number of sample coughing episodes. Second, the data were based on a model. Clinical studies on the reduction of aerosolization during upper endoscopy using our novel mask are warranted.

The prevalence of COVID-19 is not likely to decrease in the future. Therefore, simple and effective infection control measures are essential for those who perform regular endoscopic procedures. When used in combination with personal protective equipment, the novel mask may effectively reduce transmission via microdroplets.

## Acknowledgments

The authors are grateful to Mr Motohiro Mitamura (Olympus Co.) for helping in the designing of the mask using a 3D printer. We would like to thank Mr Ryuta Okamoto and Mr Kozou Takahashi (Shin Nippon Air Technologies CO., LTD.) for providing us with a high-definition camera and cleanroom.

## Author contributions

**Conceptualization:** Tadateru Maehata.

**Data curation:** Tadateru Maehata, Hirofumi Kiyokawa, Yoshinori Sato.

**Formal analysis:** Yoshinori Sato, Kazunari Nakahara.

**Investigation:** Hiroshi Yasuda, Hirofumi Kiyokawa, Hiroyuki Yamamoto.

**Methodology:** Tadateru Maehata, Masaki Yamashita.

**Resources:** Masaki Yamashita.

**Supervision:** Hiroshi Yasuda, Yasumasa Matsuo, Kazunari Nakahara, Hiroyuki Yamamoto, Fumio Itoh.

**Validation:** Hiroshi Yasuda, Yoshinori Sato, Yasumasa Matsuo, Kazunari Nakahara, Fumio Itoh.

**Writing – original draft:** Tadateru Maehata.

**Writing – review & editing:** Hiroshi Yasuda, Hiroyuki Yamamoto, Fumio Itoh.
